# GeneFriends: An online co-expression analysis tool to identify novel gene targets for aging and complex diseases

**DOI:** 10.1186/1471-2164-13-535

**Published:** 2012-10-06

**Authors:** Sipko van Dam, Rui Cordeiro, Thomas Craig, Jesse van Dam, Shona H Wood, João Pedro de Magalhães

**Affiliations:** 1 Integrative Genomics of Ageing Group, Institute of Integrative Biology, University of Liverpool, Liverpool, L69 7ZB, UK; 2 Laboratory of Systems and Synthetic Biology, Wageningen University, Wageningen, 6703 HB, Netherlands

**Keywords:** Aging, Cancer, Functional genomics, Mitochondrial disease, Network biology

## Abstract

**Background:**

Although many diseases have been well characterized at the molecular level, the underlying mechanisms are often unknown. Nearly half of all human genes remain poorly studied, yet these genes may contribute to a number of disease processes. Genes involved in common biological processes and diseases are often co-expressed. Using known disease-associated genes in a co-expression analysis may help identify and prioritize novel candidate genes for further study.

**Results:**

We have created an online tool, called GeneFriends, which identifies co-expressed genes in over 1,000 mouse microarray datasets. GeneFriends can be used to assign putative functions to poorly studied genes. Using a seed list of disease-associated genes and a guilt-by-association method, GeneFriends allows users to quickly identify novel genes and transcription factors associated with a disease or process. We tested GeneFriends using seed lists for aging, cancer, and mitochondrial complex I disease. We identified several candidate genes that have previously been predicted as relevant targets. Some of the genes identified are already being tested in clinical trials, indicating the effectiveness of this approach. Co-expressed transcription factors were investigated, identifying C/ebp genes as candidate regulators of aging. Furthermore, several novel candidate genes, that may be suitable for experimental or clinical follow-up, were identified. Two of the novel candidates of unknown function that were co-expressed with cancer-associated genes were selected for experimental validation. Knock-down of their human homologs (C1ORF112 and C12ORF48) in HeLa cells slowed growth, indicating that these genes of unknown function, identified by GeneFriends, may be involved in cancer.

**Conclusions:**

GeneFriends is a resource for biologists to identify and prioritize novel candidate genes involved in biological processes and complex diseases. It is an intuitive online resource that will help drive experimentation. GeneFriends is available online at: http://genefriends.org/.

## Background

Over the last decade, microarray technology has allowed researchers to measure gene expression levels across large numbers of genes simultaneously, identifying genes and biological processes that are activated or suppressed under different conditions. Potential biomarkers [1-4] and genes involved in a number of diseases, such as cancer, have been identified by microarray analyses [5,6]. By combining gene expression data in a meta-analysis, greater power and more information can be gained from existing data. Meta-analyses have been successfully used to identify new relationships between genes and new candidate disease-associated genes [7,8]. Microarrays provide large-scale, genome-wide data, from which coordinated changes in gene expression can be inferred. These coordinated changes are valuable in understanding the factors involved in disease and the functions of many poorly studied genes. One of the issues that arises with these large-scale datasets, however, is that it becomes harder to interpret the data and identify key players. For this reason we created a tool to facilitate this process: GeneFriends.

GeneFriends is based on a co-expression analysis, in which the general behaviour of genes relative to each other is studied. This makes it possible to uncover genetic modules that are functionally related [9], under the assumption that those genes active in the same biological processes are co-expressed. The main theory behind this approach is that functionally related genes are more likely to be co-expressed [10-12]. This “guilt-by-association” concept has already been used to relate hundreds of unidentified genes to inflammation, steroid-synthesis, insulin-synthesis, neurotransmitter processing, matrix remodelling and other processes [7,13]. Some of the predicted results have been experimentally validated demonstrating the effectiveness of this approach [7]. Candidate genes for cancer, Parkinson’s and Schizophrenia have also been identified using this approach [1,13-15]. Furthermore, it is possible to identify transcriptional modules that may play causative roles in the disease or process under study [7,8].

The aim of this work was to construct an online tool that can be used to derive novel candidate genes for further studies in aging and complex diseases, in a quick and intuitive manner. Aging is not considered a disease, yet older individuals are more susceptible to several diseases such as Alzheimer’s, Parkinson’s and cancer. This is one of the reasons why research in this field is rapidly expanding and several hundreds of genes have been linked to aging [16]. A major bottleneck in aging/complex disease research is that it is difficult to determine the causality of transcriptional alterations. It is also unclear if the altered expression profile observed with aging/complex disease consists of one particular biological module or whether it consists of genes that act separately from each other. To this end, GeneFriends outputs transcription factors co-expressed with the genes supplied by the user.

Underlying GeneFriends is a genome-wide co-expression map created using over 1,000 mouse microarray datasets. We validated our co-expression map by showing that functionally related genes are more likely to be co-expressed. We then used GeneFriends to study transcriptional changes with aging, cancer and mitochondrial disease. Multiple candidate genes associated with cancer and mitochondrial diseases, including un-annotated and poorly studied genes, were identified. Two of the novel candidates of unknown function that were co-expressed with cancer-associated genes were experimentally validated by knock-down in HeLa cells which slowed growth, supporting our predictions. This demonstrates that GeneFriends is a useful resource for studying complex diseases/processes and can infer function of poorly studied genes. GeneFriends is freely available online to allow researchers to quickly identify candidate genes co-expressed with their genes of interest (http://genefriends.org/).

## Results

### GeneFriends: an online tool for the research community

The aim of the project was to create a user-friendly tool, which can take a list of genes related to a given disease or process and quickly identify new candidate genes. Using co-expression profiling the genes are given in rank order to help prioritize candidates for experimental follow up. Underlying GeneFriends is a *Mus musculus* co-expression map created from 1,678 microarray datasets, comprising over 20,000 individual samples from previously published experiments. To create the co-expression map we employed a vote counting method. The co-expression map contains ≈ 427.5 million gene pairs (20,676 x 20,676) arranged in a matrix and given a score based on how often they are co-expressed across all microarrays (see Materials and Methods).

The input for GeneFriends is either a single gene or a list of Entrez or gene symbol identifiers. The output is a simple, clear list of co-expressed genes, which can be downloaded or viewed online. GeneFriends has the following functionalities:

1. It searches for co-expressed genes based on a seed list or a single gene, and provides a ranked list of significantly co-expressed genes.

2. It identifies the Gene Ontology (GO) terms and enrichment for the significantly co-expressed genes.

3. It returns a ranked list of significantly co-expressed transcription factors.

We feel this output will help researchers in various fields identify interesting genes for follow up studies in a quick and intuitive manner. To test if this novel tool can be used to derive biologically-relevant predictions we tested gene sets related to aging, cancer or mitochondrial complex I disease. Furthermore, we tested two predicted novel candidates experimentally, as detailed below.

### Testing the co-expression map

**Figure 1:**
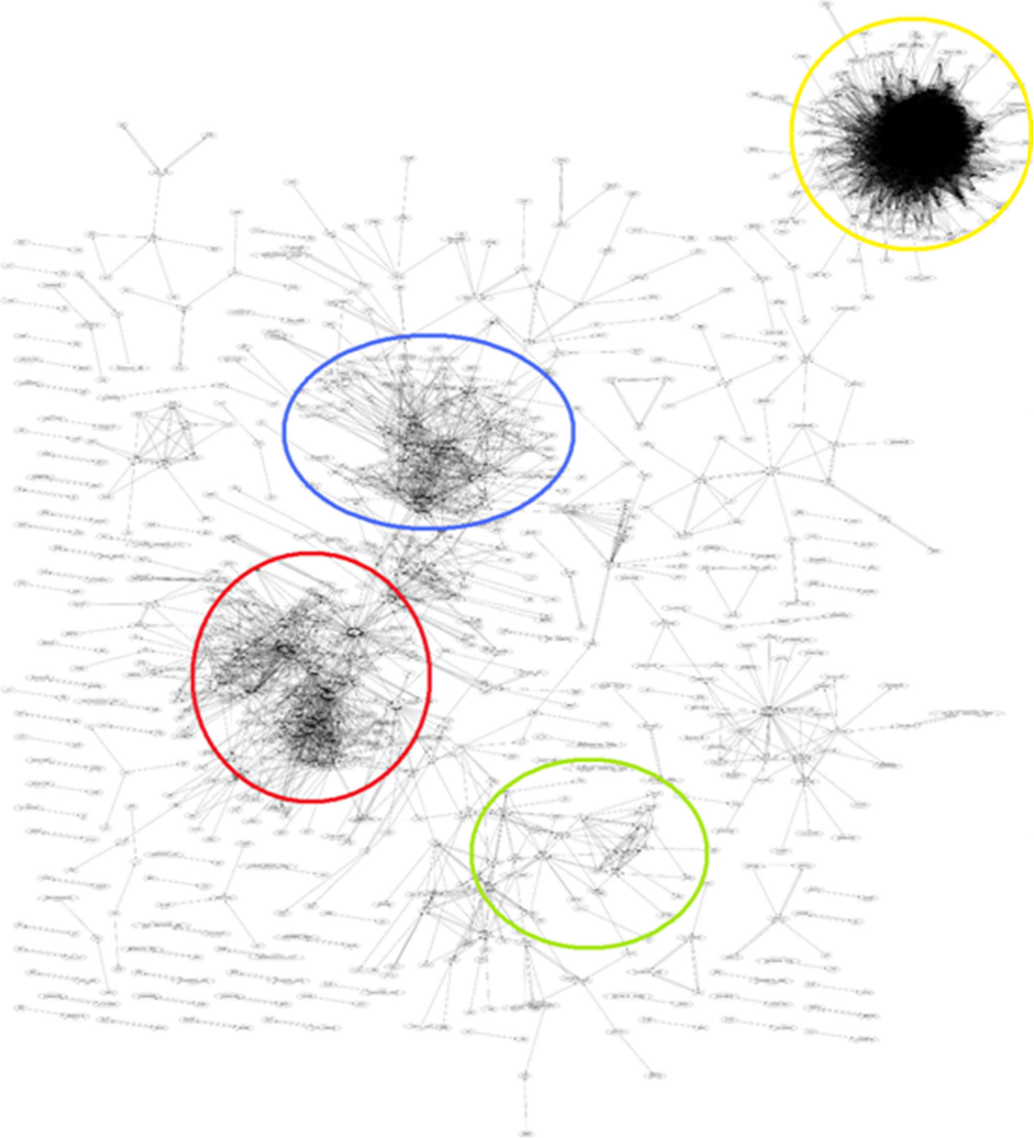
**Gene clustering in the network of co-expressed genes.** Each node represents a gene, and connections indicate a co-expression ratio of at least 80% between gene pairs. Yellow: Cell cycle (Enrichment score: 66, Benjamini: 5.2x10^-83^); Blue: Extra-cellular matrix, collagen( Enrichment score: 16, Benjamini: 3.0x10^-22^); Red: Immune system (Enrichment score: 6, Benjamini: 1.3x10^-16^); Green: Fatty acid metabolism (Enrichment score: 16, Benjamini: 1.6x10^-23^).

The biological significance of the co-expression map was verified using nine genes known to be involved in three biological processes: cell division cycle, immune response and fatty acid metabolism. The top 5% of genes identified by GeneFriends as co-expressed were analysed by DAVID [17] to detect enriched biological processes and functions. The results confirmed that genes co-expressed were functionally related to either cell cycle (Enrichment score: 56.5, FDR: 3.0x10^-75^), immune response (Enrichment score: 14.7, FDR: 7.8x10^-13^) or fatty acid metabolism (Enrichment score: 20.98, FDR: 1.2x10^-23^); detailed results are included in the supplementary data (Additional file 1). Furthermore, Figure 1 shows the clustering of the co-expression map’s network, demonstrating that co-expressed genes tend to be involved in the same biological processes. 

GeneFriends uses a vote-counting method to rank co-expression. We compared GeneFriends to COXPRESSdb [9], which utilizes the more commonly used correlation value (Pearson or Fisher). To do so we selected 3 genes with known functions and retrieved output from both tools and used DAVID to determine enriched categories. The results show similar categories and scores although the overlap in the specific genes can vary (Additional file 2). When comparing the numbers of transcription factors present in the top 1000 co-expressed genes from GeneFriends and COXPRESSdb the results are similar. This demonstrates that our approach is similar to using Pearson correlation to create a co-expression map.

### Candidate gene prediction from process/disease gene lists

We used GeneFriends to identify novel candidate genes associated with specific processes or diseases. The results show the numbers of times each of the 20,676 genes in the co-expression map were "friends" with genes in the disease gene seed and corresponding p-values indicate the statistical significance of the co-expression (see Materials and Methods). The p-value is calculated based on the number of seed genes a given gene is co-expressed with and the total number of genes it is co-expressed with (Materials and Methods). DAVID was used to interpret the broader biological significance of the results. All genes with a co-expression p-value <10^-6^ were classified using the default settings in DAVID. This is a stringent cut-off since using a Bonferroni correction for multiple testing results in: 0.05/20,677 > 10_._^-6^

### Aging-related gene prediction and putative transcriptional mechanisms

GeneFriends was used to identify genes related to aging. A seed list of genes known to be consistently over-expressed with age in mammals was used [18]. In total, 1119 genes were co-expressed with the aging seed list at p <10^-6^; Table 1 shows the top 25 genes. Many of these genes have been associated with age-related diseases. Several other genes that have been shown to play a role in aging such as lysosomal-associated membrane protein-2 Lamp2 [19] (p = 5.68^-30^), Fas [20] (p = 2.70^-31^) and growth hormone receptor Ghr [21] (p = 1.34^-19^) also showed a significant co-expression. Anxa2, Anxa3 and Anxa4 also show a low p-value (p < 10^-25^) as well as several S100 calcium binding proteins which have been shown to interact with annexins [22]. 

**Table 1:** Top 25 genes co-expressed with aging related genes

**Gene**	**Previous association evidence**	**Reference**
Thbs1	Plays a role in platelet aggregation, angiogenesis and tumorigenesis	[57-59]
Ctsh	No previous associations	
2310043n10rik	No previous associations	
Sat1	Induction has been suggested as a therapeutic strategy for treating colorectal cancer	[60]
Tcn2	No previous associations	
Pgcp	No previous associations	
D12ertd647e	No previous associations	
Cd74	Initiates signalling leading to cell proliferation and survival	[61]
B2m	B2m deficient mice suffer from tissue iron overload	[62]
Tgm2	Overexpression increases apoptosis in neuroblastoma cells	[63]
	Implicated in fibrosis, neurodegenerative and celiac disease	[64]
Rarres2	No previous associations	
Anxa1	Plays an important role in anti-inflammatory signalling, apoptosis and proliferation	[65,66]
Il10rb	No previous associations	
Ctsc	Mutations cause Papillon-Lefevre Disease	[67,68]
Lipa	Mutations can cause Cholesteryl ester storage disease and Wolman disease	[69]
IL3ra1	No previous associations	
Lgals3bp	Associated with cancer and metastasis	[70]
Pros1	Associated with Thrombosis	[71,72]
Fcgr2b	No previous associations	
Scd1	Plays an important role in body weight regulation and development of obesity	[73]
Ifi35	No previous associations	
Ctla2b	No previous associations	
Cebpd	Implicated in adipocyte differentiation, learning and memory, mammary epithelial cell growth control.	[74-76]
	Loss of Cebpd leads to chromosome instability	[77]
Fcgrt	No previous associations	
H2-t23	No previous associations	

For a full list refer to Additional file 3.

The most significantly over-represented functional clusters were inflammatory response (enrichment score (ES) = 24.13, FDR = 1.97x10^-18^), vasculature development (ES = 10.18, FDR = 2.31x10^-8^) and lysosome (ES = 9.00, FDR = 2.25x10^-8^). Since most of the genes in the seed list were classified in the categories related to the immune system, it was unsurprising to find similar results for the co-expressed genes.

Eighty genes from the initial 181 genes in the aging seed list showed a co-expression p-value <10^-6^, suggesting the presence of shared transcriptional modules. In order to investigate the underlying transcriptional mechanisms that may induce this expression profile we used the co-expressed transcription factor (TFs) results from GeneFriends. Table 2 shows the 20 most significantly co-expressed TFs with aging. The most significant TFs identified were C/ebpα, C/ebpβ and C/ebpδ (Table 2). Interestingly, these TFs show co-expression (i.e., in top 5% of co-expressed genes) with a significant proportion of the genes co-expressed with the aging seed list: 477 out of 1119 genes (p-value < 10^-100^) for all 3 TFs and 730 out of 1119 (p-value < 10^-100^) were co-expressed with at least 2 out of 3 C/epβ genes.

**Table 2:** Top 10 co-expressed transcription factors with genes increased in expression with aging

**Transcription factor**	**p-value**	**Gene Name**
C/ebpδ	7.90x10^-34^	CCAAT/enhancer binding protein (C/EBP), delta
C/ebpα	1.19x10^-30^	CCAAT/enhancer binding protein (C/EBP), alpha
C/ebpβ	3.78x10^-30^	CCAAT/enhancer binding protein (C/EBP), beta
Creg1	1.70x10^-29^	cellular repressor of E1A-stimulated genes 1
Nfe2l2	1.17x10^-28^	nuclear factor, erythroid derived 2, like 2
Irf7	8.04x10^-26^	interferon regulatory factor 7
Klf2	1.86x10^-23^	Kruppel-like factor 2 (lung)
Irf1	8.17x10^-23^	interferon regulatory factor 1
Ostf1	1.96x10^-22^	osteoclast stimulating factor 1
Atf3	2.09x10^-22^	activating transcription factor 3

Since these TFs are co-expressed with the aging-related genes it was expected that these genes, at least in part, would be regulated by the co-expressed TFs. Therefore, they would share transcription factor binding sites (TFBS) for these TFs. To identify over-represented binding motifs in the genes co-expressed with the aging genes (p-value < 10^-6^) we employed factorY [23]. For the aging gene set this revealed Nfkb as the most significant result (Additional file 4). Some of the TFBS identified have co-expressed genes with the aging seed list such as NFKB1 (p_TFBS_ =1.48 x10^-5^, p_Coexpress_ = 4.44 x10^-9^), the C/ebp (p_TFBS_ = 6.95 x 10^-3^, p_Coexpress_ =7.9 x 10^-34^) genes and IRF1 (p_TFBS_ = 5.8 x 10^-4^, p_Coexpress_ =8.17x10^-23^) genes (Additional file 4). However, TFBS for Isre, Nfkb2 (p65) and Sp1 were identified as over-represented but not co-expressed and many co-expressed TFs did not have over-represented binding sites.

### Cancer-related gene prediction

A list of 45 cancer-related genes was used as seed list for GeneFriends (see Materials and Methods). DAVID analysis identified Cell cycle (ES = 58.84, FDR = 2.9x10^-77^) and DNA replication/repair (ES = 34.99, FDR = 6.0x10^-51^) as the most significant over-represented categories for cancer-related co-expressed genes. This is expected given the fact that cancer arises from the uncontrolled division of cells. Table 3 shows the top 10 co-expressed genes.

**Table 3:** Top 10 genes co-expressed with cancer-related genes

**Gene**	**Previous association evidence**	**Reference**
Nfkbil2	Confers resistance to DNA damaging agents and is a component of the replication stress control pathway	[78]
Chtf18	Involved in checkpoint response and chromosome cohesion	[79]
Cdc25c	Over expression associated with poor prognosis of cancer	[40]
Cdc7	Effective in inhibition of cancer growth	[80]
E130303b06rik	No previous associations	
Cep152	Involved in centriole duplication	[25]
Bc055324	No previous associations	
Cenpp	Required for proper kinetochore function and mitotic progression	[81]
Anln	Increased in expression in lung carcinogenesis and suggested as target	[82]
Hirip3	No previous associations	

For a full list refer to Additional file 5.

From the original seed list only 6 genes pass the p <10^-6^ threshold co-expressed with the entire set of cancer genes, which could be due to the heterogeneity of cancer etiology. However, there were several significantly co-expressed genes, not included in the seed list, that have previously been associated with cancer. For example, Cdc25a, Cdc25b and Cdc25c, members of the Cdc25 family, are significantly co-expressed (p < 10^-6^) with the cancer-related genes.

There were a high number of significantly co-expressed centromeric proteins co-expressed with the cancer seed list. These proteins play a role in chromosome segregation and incorrect segregation of chromosomes during the cell cycle can lead to cancer [24]. Cep152 is involved in centriole duplication [25]. Cenpp, as well as CenpN/F/H/J/I/C1/T/K/M/E/Q/A/L, are all co-expressed significantly with the cancer seed list (Additional file 5) and are part of the Cenp-A NAC complex. This complex is required for proper kinetochore function and mitotic progression and its disruption can lead to incorrect chromosome alignment and segregation that preclude cell survival despite continued centromere-derived mitotic checkpoint signalling [26,27]. Plk1, Aurka, Aurkb and Cdca8 are in the top 50 co-expressed genes, these play an important role in cancer formation [28,29].

Several un-annotated genes (Bc055324, E130303B06Rik, 4930547N16Rik, F730047E07Rik, 1110034A24Rik, and 4632434I11Rik) were co-expressed with the cancer-related genes, suggesting these genes might play a role in occurrence or pathophysiology of cancer. One of these un-annotated genes, Bc055324, is a predicted protein coding gene, which has a high co-expression ratio of more than 0.7 with the cancer genes Rad51 and Ccdc6 [30], indicating this gene is increased in expression in >70% of the cases when Rad51 is increased in expression. Many other cancer-related genes such as Brca1 and Brca2 also show a strong co-expression with the Bc055324 gene (Additional file 6). All genes co-expressed with Bc055324 show enrichment for the cell cycle (ES = 52, FDR = 1.7x10^-74^). A BLAST analysis of the protein sequence shows no significant homology to other *Mus musculus* proteins. Similar sequences, however, are found in a large number of different multi-cellular species such as *Gallus gallus, Bos taurus* and *Homo sapiens* and there also is a significantly similar gene present in *Arabidopsis thaliana*, suggesting it is conserved in plants as well, indicating it plays a functional role.

#### Validating the role of C1ORF112 and C12ORF48 in growth of cancer cells

**Figure 2:**
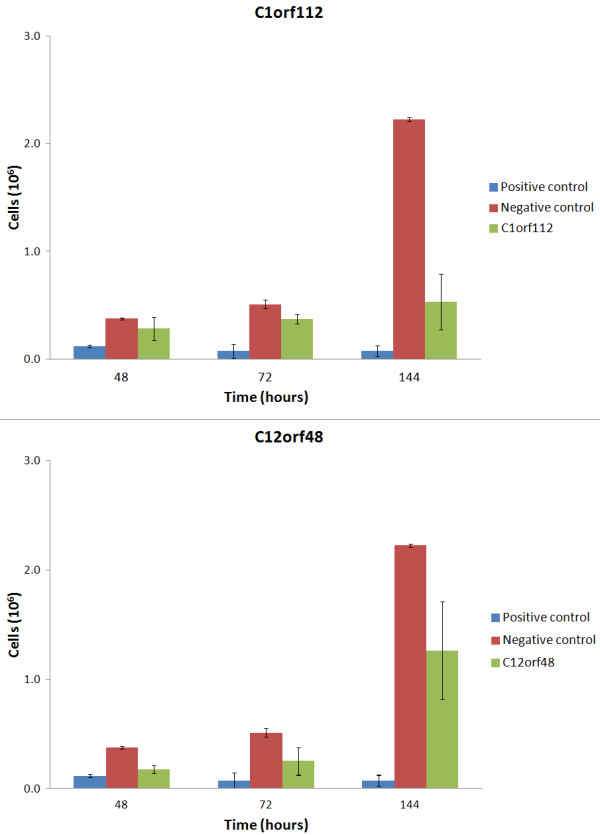
**Knock-down of candidate cancer related genes slows growth of HeLa cells.a**. Cell counting assay for the knock down of the human homolog gene of Bc055324 (C1ORF112). **b**. Cell counting assay for the knock down of the human homolog gene of 4930547N16Rik (C12ORF48). Error bars indicate the standard deviation. Negative control contains siRNA's targeting non-mammalian genes. Positive control contains siRNA's inducing apoptosis

To test our predictions, we employed siRNAs to knock down the human homologs of Bc055324 (C1ORF112) and 4930547N16Rik (C12ORF48) in the widely-used HeLa cancer cell line. These two genes were selected for validation because they are co-expressed with genes involved in the cell cycle (Additional file 6), so knockdown should lead to a measurable phenotype. Furthermore, validated siRNAs are available (see Materials and Methods) for these genes. The results show that the growth rate of the cancer cells is significantly lower when either C1ORF112 or C12ORF48 are knocked down (Figure 2). These results support our predictions and demonstrate that C1ORF112 and C12ORF48 are functional.

### Mitochondrial complex I disease-related gene prediction

All 10 genes in the seed list of mitochondrial complex I disease genes were significantly co-expressed with each other. This was the strongest co-expression amongst all disease gene seed lists tested, indicating they are involved in the same process and are tightly regulated (Additional file 7). Table 4 shows the top 10 co-expressed genes with the seed list. The results included a number of genes that have been associated with several diseases amongst which Alzheimer’s and Parkinson’s disease. Not surprisingly, DAVID analysis identified, Mitochondrion (ES = 210.25, FDR = 3.6x10^-250^), Cellular respiration (ES = 18.25, FDR = 5.9x10^-23^) and Oxidoreductase activity, acting on NADH (ES = 14.49, FDR = 2.3x10^-22^), as the most significant functional clusters.

**Table 4:** Top 10 genes co-expressed with mitochondrial complex I disease related genes

**Gene**	**Previous association evidence**	**Reference**
Atp5j	Risk factor for ischemic heart disease end-stage renal disease	[83]
Cox7a2	No previous association evidence	
Ndufa1	No previous association evidence	
Ndufb7	No previous association evidence	
Colx7c	No previous association evidence	
Cox5b	Interacts with the human androgen receptor	[84]
Atp5f1	No previous association evidence	
D830035I06/Atp5k	Atp5k has been associated with atherosclerosis	[85]
Deb1	*C.elegans* mutants were paralyzed and had disorganized muscle	[86]
Ndufb6	No previous association evidence	

For a full list refer to Additional file 7.

The co-expressed genes include several mitochondrial complex I genes (not in seed list), multiple cytochrome c proteins and genes involved in the ATP synthase complex. Furthermore, there are approximately 50 poorly/un-annotated genes co-expressed. A pseudo gene, 3000002C10Rik, shows a co-expression ratio of >0.5 with 512 genes. Classification of these 512 genes using DAVID results in an enrichment score of 53.7 (FDR = 2.9x10^-70^) for mitochondrial genes. Therefore, 3000002C10Rik may play a biologically relevant role in mitochondrial processes.

### Predicting functions of un-annotated genes

To investigate if it is possible to predict or estimate a given gene’s function based on its co-expression pattern we inspected a selection of un-annotated genes. Using DAVID, the functional categories for the top 5% co-expressed genes were obtained. Table 5 shows the functional categories for the un-annotated genes with the highest significance value. While some of the categories identified are broad, others are more specific. Together with the results for C1ORF112 or C12ORF48, these results show that it is possible to use GeneFriends to infer gene function.

**Table 5:** Top functional annotation clusters of the 5% strongest co-expressed genes with un-annotated genes

**Un-annotated Gene**	**DAVID Functional Annotation**	**ES**	**FDR**
0610006I08Rik			
	Mitochondrion	32.75	1.1x10^-40^
0610006L08Rik			
	Disulfide bond/secreted	33.14	3.1x10^-38^
	PeptidaseS1/Chymotrypsin	16.25	1.4x10^-20^
0610010D20Rik			
	Peroxisome	21.8	3.8x10^-22^
	Fatty acid metabolism	21.78	1.7x10^-22^
	Drug metabolism/CytochromeP450	17.94	6.1x10^-20^
0610031J06Rik			
	Lysosome	18.59	2.4x10^-18^
0610037L13Rik			
	Ribosomal protein	16.97	1.2x10^-22^
0610037M15Rik			
	Immune response	24.02	2.0x10^-28^
0710008K08Rik			
	Vasculature development	13.26	1.9x10^-12^
	Lung development	12.36	2.4x10^-10^

Clusters from DAVID with an enrichment score (ES) above 5 are displayed (10 for 0610010D20Rik). Cluster titles and FDR were selected based on the most significant annotation within the cluster. For full lists refer to Additional file 8.

## Discussion

### GeneFriends: a genetics and genomics tool for the research community

Genefriends is freely available online (http://genefriends.org/) and is an intuitive tool, which can be used to identify the genes co-expressed from a user supplied gene list. This simple, yet powerful new tool can be a valuable resource for genome interpretation, annotation, mouse genetics, functional genomics and transcriptional regulation. It may also be useful to develop network analyses of mouse genes in a variety of studies.

We tested GeneFriends to determine whether it can give biologically relevant data. We also demonstrated how GeneFriends can be used to quickly identify interesting gene targets for follow up studies. Furthermore, we experimentally validated two un-annotated genes co-expressed with a cancer seed list. Below we discuss the findings we have obtained from our example analyses and the biological relevance of our results.

### Validation of the co-expression map

The main assumption made when constructing our mouse co-expression map is that co-expressed genes tend to be involved in the same biological processes. Our results clearly support this, showing that genes known to play a role in certain biological processes such as immune system, cell cycle and lipid metabolism are co-expressed and form clusters in the co-expression network (Figure 1). Different clusters show different strengths of co-expression, therefore the effectiveness of the gene set analyses may vary. However, there is a high degree of functional coherence between co-expressed genes showing that our co-expression map can be used to obtain biologically-relevant information.

Given the intrinsic noisy nature of microarray data, we used a vote counting approach, which is a standard meta-analysis technique, to build our co-expression map [31]. The aim of this approach is to emphasize sensitivity. This is particularly important when combining large and diverse datasets and has been shown to increase sensitivity when studying aging, for example [18]. From a technical point of view, we aimed to include as much data as possible and because of the technical implications accompanied by this the vote counting method is more efficient and has faster run times than other methods.

### Co-expression analysis of genes associated with aging

Using a guilt-by-association method we identified candidate genes related to seed lists of genes associated with diseases or processes. Our study not only identified genes relevant to current theories of aging, e.g. inflammation, but it also identified novel candidates for further research. C/ebp transcription factors showed the strongest co-expression and are therefore candidate activators of the altered expression patterns with age. TFBS for C/ebp genes were identified in the aging genes and there is some evidence of a transcriptional cascade via SP1 [32]. The two proteins encoded by the C/ebpβ gene are liver activating protein (LAP) and liver inhibiting protein(LIP), which have opposing effects [33,34]. The LIP protein is also capable of inhibiting other C/ebp proteins. This could explain why C/ebp transcription factors themselves are not found to be increased/decreased in expression with age. This could also be due to the fact that TFs are sometimes expressed at low levels not detected by microarrays.

Replacement of the C/ebpα gene with C/ebpβ increases lifespan by 20% [35,36], and may alter the rate of aging [37], indicating that altering the isoform expression of these genes can affect lifespan. Moreover, the life-extending drug rapamycin may affect isoform ratios of C/ebpβ. Rapamycin has been shown to increase lifespan via the suppression of Mtor [38] which in turn controls the isoform ratios of C/ebpβ [39]. Therefore, we speculate that rapamycin may in part exert its life extending effect through C/ebpβ.

### Co-expression analysis of cancer genes and experimental validation of candidates

We used GeneFriends to identify new candidate genes for a role in cancer. Many of the cancer genes in the initial seed list were not present in the results, indicating they are not co-expressed with each other. This may be due to the fact that this set of cancer genes includes both oncogenes and tumour suppressor genes which are not expected to be co-expressed. Also cancer can arise through different mechanisms. Therefore the genes identified as co-expressed in this study are likely involved in common pathways leading to cancer, or are at least triggered by transformation.

Genes that are co-expressed with several oncogenes may prove to be useful targets in countering the proliferating effect of these genes in tumours. Examples of such genes that are already being studied are Cdc7 and Cdc25 genes, both of which were identified as co-expressed in our study. Cdc25 has been suggested as a therapeutic cancer target and on-going studies in this direction have shown some level of success [40-43]. Two compounds that target Cdc7 are currently in phase I clinical trials [44]. The fact that candidate genes identified by our method have already been suggested as potential drug targets shows that GeneFriends can be useful for the identification of candidate targets for cancer studies.

Bc055324 is one of the poorly annotated genes that is strongly co-expressed with a large number of cancer genes. Knock-down of the human homolog, C1ORF112, in HeLa cells diminishes cell growth, which adding the fact that Bc055324 knockout mice are not viable [45] (http://www.europhenome.org/), demonstrates that this gene is functional. Further studies of this gene in the context of cell cycle regulation, development and cancer are warranted. These results show that GeneFriends can indeed be used to identify novel targets for particular diseases. In addition it confirms that the functional enrichment of co-expressed genes can give indications about an un-annotated gene's function. The other un-annotated gene co-expressed with cancer we tested was 4930547NRik (C12ORF48). C12ORF48 was recently shown to be over-expressed in pancreatic ductal adenocarcinoma cells [46] and in other aggressive and therapy-resistant malignancies [46]. In line with our findings in HeLa cells, knock down of C12ORF48 significantly suppressed PDAC cell growth [46].

### Co-expression analysis of mitochondrial I complex disease genes

Mitochondrial complex I diseases include isolated complex I deficiency, which is the most common enzymatic defect of the oxidative phosphorylation disorders and can cause a wide range of clinical disorders [47,48]. These include macrocephaly with progressive leukodystrophy, nonspecific encephalopathy, cardiomyopathy, myopathy, liver disease, Leigh syndrome, Leber hereditary optic neuropathy, and some forms of Parkinson’s disease [49-51]. Mutations in the nuclear encoded mitochondrial genes have been associated with several pathologies [52,53]*.* However, half of the patients with mitochondrial complex I (CI) deficiencies lack mutations in any known CI subunit suggesting that yet unidentified genes crucial for maturation, assembly, or stability of CI may be involved in these diseases [53]. We identified several un-annotated genes that show a strong co-expression with the mitochondrial disease gene set. As most of the other co-expressed genes encode mitochondrial proteins, these un-annotated genes most likely also encode mitochondrial proteins. This is further supported by the fact that a number of these un-annotated genes have been shown to be active in the mitochondria in another large-scale study [54]. Some of these genes could be responsible for the CI deficiency phenotype and are therefore promising candidates for further studies.

## Conclusions

In this study we created a tool that identifies co-expressed genes from a user’s seed list. Moreover, it returns the GO term enrichment of this list as well as a separate list of the co-expressed transcription factors. This allows novel candidate genes to be quickly identified for follow up studies. GeneFriends employs a biologically-relevant co-expression map and a guilt-by-association method to identify novel candidate genes for complex diseases/processes. We demonstrated the biological relevance of this tool by analysing aging, cancer and mitochondrial I complex deficiency seed lists. Furthermore, we experimentally validated two un-annotated candidate genes co-expressed with cancer-related genes. We also demonstrated how GeneFriends can be used to investigate transcription factors that are co-expressed with seed genes of interest, helping to elucidate the regulatory mechanisms. GeneFriends is freely available online (http://genefriends.org) for other researchers to identify and prioritize candidate genes to study other complex diseases and processes.

## Methods

### Data selection

To create the co-expression map, normalized microarray data obtained from the GEO database was used [55]. GEO files GSE1 to GSE18120 were downloaded containing 16,916 datasets in total. From these, 3,850 *Mus musculus* datasets containing 64,849 microarrays and the corresponding annotation files were extracted. As mouse experiments are generally better controlled than human studies and there is less variation caused by genotypic and environmental factors in the mouse, *Mus musculus* data was chosen over *Homo sapiens* to reduce noise. Using mouse data also allows more datasets, coming from a more diverse set of experiments [18]. This also potentially allows for the investigation of target genes in the different mouse models of aging and complex diseases.

All datasets containing annotation files that did not include gene symbols for at least 90% of the probes present in the data were removed. All microarray datasets containing values higher than 25 were log transformed, under the assumption this data was non-log-transformed data. To remove poor signal, low quality or nonsense values up to 10^99^ datasets containing no values above 2log(5,000) or one or more values over 2log(20,000,000) were removed. Datasets with no reference to any annotation file were removed. Even though it is not feasible for us to perform a comprehensive evaluation of the quality of the data in each experiment, a meta-analysis is in its essence a technique to eliminate poor quality data and hence we are confident that there are no systematic errors in our analysis that artificially originate false results. After these steps 1,678 datasets containing 8,417 different conditions and 21,744 individual samples remained. The probe IDs were converted into gene symbols. If multiple probe IDs mapped to the same gene symbol, they were averaged. Within each dataset the experimental conditions were manually determined. Microarrays from individuals under the same conditions were averaged; in other words, duplicates were averaged. Missing values were ignored as long as there were duplicates; if there were no duplicates this gene symbol was removed.

### Constructing the co-expression map

To create GeneFriends we first constructed a genome-wide co-expression map, using normalized *Mus musculus* microarray data from the GEO database. This describes which genes are related based on how often they are co-expressed. In total, 1,678 mouse datasets containing 8,417 different conditions and 21,744 individual samples met our data selection criteria. To construct our expression map the different conditions within each dataset were compared to each other. Since different datasets contain different probes mapping to different gene symbols a selection was made. Only those gene symbols that are present in gene platform file GPL1261 (Affymetrix GeneChip Mouse Genome 430 2.0 Array) were used. This platform contains 20,676 gene symbols and is the most common platform used for microarrays amongst those included in this work. All of these gene symbols were present in over 850 datasets.

In this work we have used a vote counting approach to quantify co-expression for approximately 400 million (20,676*20,676) gene pairs. We used these pairs to establish if genes were co-regulated; co-regulation being defined as both genes increasing or decreasing in expression at least two-fold simultaneously, a standard (even if arbitrary) measure of differential expression. Then based on how often gene pairs were co-regulated compared to how often the single genes showed a two-fold increase or decrease in expression we calculated a co-expression ratio, which quantifies how strongly two genes are co-expressed, for all 20,676*20,676 gene pairs. The number of times two genes were simultaneously differentially expressed in the same direction (i.e. relative up or down regulated) was calculated using the equation:

(none1) $$\begin{array}{c} Ngene1,\;gene2=\sum_{i=0}^{x}UPgene1,\;\text{i}UPgene2,i\;\\ \;+\sum_{i=0}^{x}DOWNgene1,\;DOWNgene2,i \end{array}$$

Where *x* is the total number of comparisons and N the number of times two genes are differentially expressed (in the same direction) simultaneously.

For simplification the terms “UP” and “DOWN” are used in this formula. The actual directionality of the change is irrelevant as it is dependent on the direction of the comparison (i.e. whether one compares group 1 with group 2 or the other way around). Either way the results will be the same.

To reduce the effects of noise present in microarrays, an arbitrary two fold cut-off was selected, as is used in the majority of microarray analyses to indicate differential expression of genes. The total number of times each gene was relatively up or down regulated (i.e., >2 fold) was calculated using the following equation:

(none2) $$Qgene1\;=\sum_{i=0}^{x}DIFFERENTIALLY\_EXPRESSEDgene1$$

Where *x* is the total number of comparisons and *i* describes the current comparison between the different conditions.

From the values *N* and *Q* the co-expression ratio was deducted. The genes were then ranked based on their *N*:*Q* ratio. A ratio of 0.50 would indicate that if gene 1 is increased or decreased in expression in 50% of the cases gene 2 is also increased or decreased in expression. Each gene pair is present in at least 850 datasets and so the ratio is based on a large number of measurements.

### Testing the co-expression map

To investigate the capacity of the co-expression map to provide biologically-significant results nine genes known to play a role in specific biological processes were investigated: three genes that are known to be active in fatty acid metabolism: *Ppara, Acaa2* and *Acadm*; three genes known to be involved in immune response: *Cd4*, *Cd8* and *Il10*; and three cell cycle genes: *Cdc6*, *Cdc7* and *Cdc8*. These were selected before the analyses to reduce biases.

It was expected that genes co-expressed with these nine genes would be involved in the same biological processes; to test this assumption we inspected if any specific categories were over-represented by these groups of genes (see below for functional enrichment analysis).

### Prediction of novel candidate genes in aging and complex diseases

In order to identify genes co-expressed with known disease genes, three disease related gene sets were included. The first of these was an aging gene set. It consisted of genes over-expressed with age obtained from a meta-analysis of aging microarray studies in mice, rats and humans that revealed several conserved genes increasing or decreasing in expression with age [18] (Additional file 9). The second gene set included was a set of cancer-related genes [56] (Additional file 9). This is a manually curated cancer set that includes only heritable cancer genes with strong evidence that mutations in these genes are causative for cancer*.* The third gene set added included genes known to cause diseases through mitochondrial complex I deficiencies. The genes in this set contain the nuclear mitochondrial complex I deficiency genes in the OMIM database (Additional file 9). Gene symbols that were not present in the co-expression map were not included in the analysis.

Using the above seed lists, a "guilt-by-association" approach was employed to find new potential disease-related gene targets. In this approach the top 5% most co-expressed genes with each gene were considered “friends” of that particular gene. For each of the 20,676 genes we calculated how many times it was “friends” with the disease related genes. Next, the probability that a gene was “friends” with this number of disease genes was calculated, as follows. How often each gene was “friends” with any other gene was counted, from which the chance a gene is "friends" with another gene was calculated:

(none3) $$\begin{array}{c} p=\text{total number of friends with other genes}\\ \;/\text{total number of genes} \end{array}$$

Where *p* is in effect the chance that a particular gene occurs in the top 5% of a random gene.

We assume the following null hypothesis: The probability of a gene being a “friend” with one of the *n* disease genes equals the probability *p* of being a “friend” with a random gene. Then the probability of a gene being a “friend” with *k* or more genes from the disease list can be calculated by using the right-tail of the binomial distribution.

(none4) $$\mathrm{Pr}\left(K>=k\right)=\sum_{k=k}^{n}\left(\begin{array}{c} n\\ k \end{array}\right)p^{k}{\left(1-p\right)}^{n-k}$$

Where Pr(K > = k) is the probability that a gene would be “friends” with *k* or more genes in the disease gene set; *k* is the number disease gene “friends”; *n* is the number of genes in the gene set. When calculating *p* the number of occurrences of a gene in the top 5% of all genes was included. This is necessary since some genes tend to be co-expressed more often in general than other genes.

### Experimental validation of cancer-related genes Bc055324 and 4930547N16Rik

To test the predictions from the analyses using GeneFriends, we took un-annotated genes that were the most co-expressed with the cancer disease gene list. Validated siRNAs were available from Qiagen for two the human homologs of the top un-annotated genes: Bc055324 (C1ORF112) and 4930547N16Rik (C12ORF48). The experiment was conducted in human HeLa cells using standard culture conditions. A negative and a positive control were also included (Qiagen). The positive control contained a mix of several apoptosis inducing siRNAs, demonstrating that the transfection was successful through elevated cell death. The negative control consisted of siRNAs targeting non-mammalian genes. The full protocol followed for this experiment is described in Additional file 10.

### Gene function enrichment analysis

The Database for Annotation, Visualization and Integrated Discovery (DAVID) [17] was used to identify enriched functional groups within these genes. The default settings were used in this analysis. The results were ranked based on p-value and genes with a p-value <10^-6^ were selected. This is a stringent cut-off since using a Bonferroni correction for multiple testing results in: 0.05/20,677 > 10^-6^_._ In addition, a randomization test showed this is a very stringent cut-off in which no false positives are expected by chance. The test entailed the construction of several random sets of genes which were then used to find co-expressed genes. The p-values found for all results were >10^-5^, indicating that genes with a p-value <10^-5^ are very unlikely false positives.

Next, to understand the significance of the DAVID enrichment score, 1000 genes were randomly selected and used as an input for DAVID. This resulted in an enrichment score of 2.2 with an FDR score of 0.7 for the most significant category found. The same was done for smaller numbers resulting in similar scores. This indicates that the Enrichment scores of >10 and FDR <10^-10^ are very significant and cannot be found by random chance*.*

Benchmarking using our co-expression map and COXPRESSdb revealed similar results (Additional file 2), suggesting our co-expression map is not inferior to those built using correlation measures. Therefore, our work demonstrates that vote counting is a viable method to build co-expression maps.

One concern is that some of the genes in functional categories were assigned based on their expression pattern; if this would be the case it could lead to circular reasoning. However, an analysis conducted by the DAVID team shows that less than 1% (116/20676) of the genes is grouped based solely on their expression pattern (see Additional file 11 for a list of these genes).

### BLAST

A BLAST search was conducted on the Bc055324 gene. The protein sequence for this gene was recovered from GenBank version: Bc055324.1. This sequence was PSI-blasted against the non-redundant protein sequence database. Then the search was iterated twice including all sequences recovered in the initial search.

## Competing interests

The authors declared no conflict of interest.

## Author**s’ c**ontributions

SvD constructed and analysed the co-expression map and drafted the manuscript. JvD constructed a visual representation of the co-expression map and aided in the analyses. RC designed and conducted the experimental validation. SW edited the manuscript and aided the experimental work. TC developed the online interface, database and tools. JPdM participated in the study design, secured funding, assisted in the analyses and edited the manuscript. All authors read and approved the final manuscript.

### Funding statement

SvD thanks past support from the Erasmus programme and is funded, together with SHW, by a BBSRC grant (BB/H008497/1) to JPdM. TC is funded by a Wellcome Trust grant (ME050495MES) to JPdM. The work of RC was supported by the Erasmus programme. JPdM is also grateful for support from the Ellison Medical Foundation and from a Marie Curie International Reintegration Grant within EC-FP7. The funders had no role in study design, data collection and analysis, decision to publish, or preparation of the manuscript.

## Supplementary Material

Additional file 1 DAVID enrichment scores of 10 selected genes known to be related to cell cycle, immune response and fatty acid metabolism. Click here for file

Additional file 2 Comparison of DAVID enrichment scores of 3 genes with known functions with CoXpressDB. Click here for file

Additional file 3 Co-expressed genes with aging related gene seed list. Click here for file

Additional file 4 Co-expressed transcription factors with aging related gene seed list. Click here for file

Additional file 5 Co-expressed genes with causative cancer gene seed list. Click here for file

Additional file 6 Co-expressed genes with Bc055324 and 4930547N16Rik. Click here for file

Additional file 7 Co-expressed genes with mitochondrial complex I disease related gene seed list. Click here for file

Additional file 8 Top 5% co-expressed genes with poorly annotated genes. Click here for file

Additional file 9 Seed list containing causative cancer genes and seed list containing aging related genes. Click here for file

Additional file 10 Transfection protocol. Click here for file

Additional file 11 List of DAVID genes of which grouping is solely inferred from expression pattern. Click here for file
