# Supplementary material for: GeneFriends: An online co-expression analysis tool to identify novel gene targets for aging and complex diseases
Source: BMC Genomics. 2012 Oct 6;13:535. doi: 10.1186/1471-2164-13-535 (PMC3495651; doi:10.1186/1471-2164-13-535)
Supplement: Additional file 2 — Comparison of DAVID enrichment scores of 3 genes with known functions with CoXpressDB. [file 1471-2164-13-535-S2.docx]

**Comparison between gene enrichment for top 300 genes co-expressed with several well annotated genes with our co-expression map and CoXpressDB.** The results are mostly overlapping indicating the same or similar enrichments.

| **Gene** | **Category** | **CoXDB** | **GeneFriends** | **Overlap (genes)** |
| --- | --- | --- | --- | --- |
| **Brca1** | Number | 300 | 300 | 197 |
|  | Cell Cycle | 66.35 | 75.09 |  |
|  | Chromosome | 54.62 | 50.5 |  |
| **H2-Aa** | Number | 300 | 300 | 94 |
|  | Disulfide bond | 30.09 | 11.27 |  |
|  | Immune response | 19.5 | 14.52 |  |
| **Ppara** | Number | 300 | 300 | 100 |
|  | Fatty Acid metabolism | 8.75 | 19.1 |  |
|  | Peroxisome | 14.37 | 11.12 |  |
