# Supplementary material for: GeneFriends: An online co-expression analysis tool to identify novel gene targets for aging and complex diseases
Source: BMC Genomics. 2012 Oct 6;13:535. doi: 10.1186/1471-2164-13-535 (PMC3495651; doi:10.1186/1471-2164-13-535)
Supplement: Additional file 10 — Transfection protocol. [file 1471-2164-13-535-S10.docx]

**Transfection** (reverse) **(6-well plate)**

1. Prepare **6** 1,5mL Eppendorf tubes and to each add:
   1. **250µL** of DMEM (without antibiotics or FBS)
   2. **10µL** of 20uM siRNA
   3. **5µL** of HiPerfect Transfection Reagent;
2. Mix by vortexing;
3. Incubate for **20-45min.** at room temp. (15ᴼ-25ᴼ) to allow formation of transfection complexes;
4. Meanwhile, prepare cells for reverse transfection:
   1. Wash with PBS
   2. Trypsinize (±**500µL**)
   3. Observe under microscope to make sure that all the cells are detached from the bottom of the flask
   4. Add DMEM (without antibiotics or FBS) to inhibit trypsin reaction (±**500µL**)
   5. Count cells (Coulter Counter)
   6. Calculate the appropriate volume to pipette to obtain **2.5x10^5^** cells per well/Eppendorf Tube;
5. After incubation, pipette the appropriate volume to obtain 2.5x10^5^ cells per Epp. Tube;
6. Prepare 6-well Plates with **1mL** of DMEM with 1% FBS and antibiotics per well;
7. Transfer the contents of each Epp. Tube into one of the wells of the 6-well plates.
8. Gently swirl the plate to ensure uniform distribution of the transfection complexes.
9. Incubate cells with the transfection complexes under their normal growth conditions.
